
JOURNAL INFORMATION
==============================
Journal ID: Intereconomics

Intereconomics

EISSN: 1613-964X

ARTICLE INFORMATION
==============================
PMCID: PMC9549842
DOI: 10.1007/s10272-022-1070-5
Article ID: 1070
Article version: 1
Subjects: Editorial

The Leopard: How a Post-Fascist Party Rose to Power in Italy

Dosi Giovanni gdosi@sssup.it

Roventini Andrea aroventini@sssup.it

grid.263145.7 0000 0004 1762 600X Institute of Economics, Scuola Superiore Sant’Anna, Piazza Martiri della Libertà 33, 56127 Pisa I, Italy
Electronic publication date: 2022 Oct 10
Print publication date: 2022
Volume: 57
Issue: 5
First page: 270
Last page: 271
Copyright: © The Author(s) 2022
License: Open Access: This article is distributed under the terms of the Creative Commons Attribution 4.0 International License (https://creativecommons.org/licenses/by/4.0/).
Open Access funding provided by ZBW — Leibniz Information Centre for Economics.
License URL: https://creativecommons.org/licenses/by/4.0/

Keywords: E00, E6, O4
issue-copyright-statement: © The Author(s) 2022

==============================

Italy has been approaching general elections with deteriorating economic fundamentals. Due to the war in Ukraine, the economic recovery has slowed considerably, while the GDP is still below the 2008 level. This decade-long stagnation has hit low- and middle-income Italians harder. As a result, income and wealth inequality has been steadily increasing (Guzzardi et al., 2022). According to Eurostat, 25% of the Italian population is at risk of poverty (the EU average is 21.7%), and 11.7% of workers are poor due to temporary and part-time jobs. While employment recovered quickly in 2022, temporary jobs have skyrocketed to the record level of 3,176,000, according to ISTAT. Still in Q2 2022, inflation jumped to 6.9%, and OECD expects Italian real wages to fall by 3% over the course of 2022. This is going to further exacerbate the long-run plunge of the real wage: Italy is the only OECD member where real wages have fallen (-2.9%) since 1990.

The coronavirus pandemic and Russian war in Ukraine have magnified the fragility of the Italian economy, exposed to increasingly poisonous doses of neoliberal economic policies based on fiscal austerity and “flexibilisations” of the labour market (the Berlin–Washington consensus, see Fitoussi and Saraceno, 2013) and nested in the longer-term end of the post-war social compromise (Dosi and Virgillito, 2019). The result has been a decrease in health and education expenditures and public investment. Meanwhile, labour market structural reforms of the 1990s and 2000s have increased fixed-term and part-time contracts and raised wage inequality and volatility (Hoffman et al., 2021) with the effect of slowing down productivity and output growth (Dosi et al., 2021).

During the COVID-19 pandemic, the EU has timidly started changing its economic policy stance. With Next Generation EU (NGEU), it has issued common debt to finance a vast programme of public investment with a significant transfer of resources to its weakest members. The European Chips Act and the Fit for 55 package have possibly inaugurated a new season of innovation and industrial policy. With the directive on adequate minimum wages, the EU proposed regulation in European labour markets against a freefall to the bottom. If one is optimistic, the new EU policy framework can be seen as part of a belated general change away from the scourge of neo-liberalism (Stiglitz, 2019), including Bidenomics. Worldwide there is an increasing case for a New Consensus, grounded in more intense state intervention to tackle inequality, give workers more power, raise public expenditure in health and education, and ultimately guide the process of innovation and economic development.

A lingering question is whether the EU is actually embracing the new zeitgeist. For sure, Italy is still trapped in the adagio from the novel The Leopard: “everything must change to remain the same”. And what must not change is the neoliberal policy framework mitigated by some cosmetic corrections, witnessed both by the Italian NGEU plan and the Draghi economic policies more generally. The Italian NGEU plan is a poorly assembled collection of projects provided by the ministries often out of their longstanding list of unfunded initiatives, glued together with the buzzwords of private consultants. It is clear that the bulk of the funds will be ultimately transferred to the firms with very few conditions attached, if any. And it could have been even worse if the European Commission had not rejected the carbon-capture-and-storage project sponsored by Eni to produce blue hydrogen.

On the reform side, the Italian NGEU plan is also disappointing. The introduction of a minimum wage was jettisoned in the final version of the plan. More generally, while in other countries, e.g.

Spain, fixed-term contracts have been discouraged with good results in terms of employment, the Italian government has made them easier. The proposals of a commission created by the Ministry of Labour to tackle the issue of the working poor has been ignored, while the conditions of access to the Italian anti-poverty scheme have been made stricter. At the same time, the number of bonuses has proliferated: There are even subsides for buying new-generation televisions, anti-solar curtains and spa services. Finally, in the fiscal domain, despite evidence that Italians in the top 5% of income distribution pay a lower effective tax rate than the rest of the population (Guzzardi et al., 2022), no increase in progressivity is envisaged, rather just fine-tuning the status quo.

The fault line between Italy and any New Consensus policy agenda can be observed in climate policies. Italy has tried to postpone the introduction of the EU ban on combustion engine cars, while introducing subsidies for buying new diesel and gasoline cars. There are still generous subsidies for the purchase of gas-fired heating systems. Italy has been outpaced by Germany and the Netherlands in the installation of new solar power capacity, while the government has not succeeded in easing the bureaucratic procedures to install new renewable energy plants. However, the Minister of "Ecological Transition" has warned against the lobby of renewable energy (sic!).

The bottom line, in our opinion, is that the executive led by Mr Draghi never had a vision for Italy to achieve sustainable and inclusive growth while relaunching innovation and productivity, trapped as it largely was in the myth that "markets know better". The only opposition able to capture a good deal of social discontent has been the post-fascist right, as the Democratic Party (PD) fully identified with a wealthy and socially secure establishment.

The policy agenda of the right-wing parties fully embraces a neoliberal economic programme, which ignores the climate emergency and pushes the flat tax as its flagship policy. Conversely, the leader of the PD, Mr Letta, has tried (and failed) to form an alliance with a centrist party on the ground, excluding the Five Star Movement (M5S). Given the Italian electoral law, Mr Letta has de facto deliberately chosen to lose the elections because a coalition with the M5S was essential to win a majority of the first-past-the-post seats. At the end of the day, the M5S has been the only sponsor of a progressive agenda and performed much better than expected, while the PD confirmed its decline.

Mr Draghi recently declared that Italy shall succeed with any government. We are not as optimistic, and we see precise links between the economic policies of his government, the results of the elections and the next economic agenda. There is a more general lesson here, which goes beyond the Italian borders. When the former progressive parties give up their social agenda, the mounting fears, anger and frustration of an increasing percentage of people get picked up by right-wing movements. These movements are able to combine the continuation of neo-liberal policies in the socio-economic domain with a construction of collective identities with many fascist ascendancies: the nation, the family, the Christian roots, the hate toward the "other", the strong man/woman in charge, etc. After one century, Italy is likely to be again the victim of such a social experiment, with a (post?) fascist prime minister. We just hope our prediction is wrong.

Giovanni Dosi, Scuola Superiore Sant’Anna, Pisa, Italy.

Andrea Roventini, Scuola Superiore Sant’Anna, Pisa, Italy; and OFCE, Sciences Po, Paris, France.


References

Dosi G Freeman R B Pereira M C Roventini A Virgillito M E The impact of deunionization on the growth and dispersion of productivity and pay Industrial and Corporate Change 2021 30 377 408 10.1093/icc/dtaa025
Dosi G Virgillito M E Whither the evolution of the Contemporary Social Fabric? International Labor Review 2019 158 1 33 10.1111/ilr.12145
Guzzardi, D., E. Palagi, A. Roventini and A. Santoro (2022), Reconstructing Income Inequality in Italy: New Evidence and Tax Policy Implications from DINA, World Inequality Lab Working Paper, 2022/02.
Fitoussi J-P Saraceno F European economic governance: the Berlin-Washington Consensus Cambridge Journal of Economics 2013 37 479 496 10.1093/cje/bet003
Hoffmann, E. B., D. Malacrino and L. Pistaferri (2021), Labor Market Reforms and Earnings Dynamics: the Italian Case, IMF Working Paper, 2021/142.
Stiglitz J People, Power and Profit 2019 New York N.N. Norton
